# *Phlebotomus orientalis* Sand Flies from Two Geographically Distant Ethiopian Localities: Biology, Genetic Analyses and Susceptibility to *Leishmania donovani*

**DOI:** 10.1371/journal.pntd.0002187

**Published:** 2013-04-25

**Authors:** Veronika Seblova, Vera Volfova, Vit Dvorak, Katerina Pruzinova, Jan Votypka, Aysheshm Kassahun, Teshome Gebre-Michael, Asrat Hailu, Alon Warburg, Petr Volf

**Affiliations:** 1 Department of Parasitology, Charles University in Prague, Faculty of Science, Prague, Czech Republic; 2 Aklilu Lemma Institute of Pathobiology, Addis Ababa University, Addis Ababa, Ethiopia; 3 Department of Microbiology, Immunology & Parasitology, Faculty of Medicine, Addis Ababa University, Addis Ababa, Ethiopia; 4 Department of Microbiology and Molecular Genetics, The Institute for Medical Research Israel-Canada, The Kuvin Centre for the Study of Infectious and Tropical Diseases, The Hebrew University - Hadassah Medical School, Jerusalem, Israel; National Institutes of Health, United States of America

## Abstract

**Background:**

*Phlebotomus orientalis* Parrot (Diptera: Psychodidae) is the main vector of visceral leishmaniasis (VL) caused by *Leishmania donovani* in East Africa. Here we report on life cycle parameters and susceptibility to *L. donovani* of two *P. orientalis* colonies originating from different sites in Ethiopia: a non-endemic site in the lowlands - Melka Werer (MW), and an endemic focus of human VL in the highlands - Addis Zemen (AZ).

**Methodology/Principal Findings:**

Marked differences in life-cycle parameters between the two colonies included distinct requirements for larval food and humidity during pupation. However, analyses using Random Amplified Polymorphic DNA (RAPD) PCR and DNA sequencing of cytB and COI mitochondrial genes did not reveal any genetic differences. F1 hybrids developed successfully with higher fecundity than the parental colonies. Susceptibility of *P. orientalis* to *L. donovani* was studied by experimental infections. Even the lowest infective dose tested (2×10^3^ per ml) was sufficient for successful establishment of *L. donovani* infections in about 50% of the *P. orientalis* females. Using higher infective doses, the infection rates were around 90% for both colonies. *Leishmania* development in *P. orientalis* was fast, the presence of metacyclic promastigotes in the thoracic midgut and the colonization of the stomodeal valve by haptomonads were recorded in most *P. orientalis* females by day five post-blood feeding.

**Conclusions:**

Both MW and AZ colonies of *P. orientalis* were highly susceptible to Ethiopian *L. donovani* strains. As the average volume of blood-meals taken by *P. orientalis* females are about 0.7 µl, the infective dose at the lowest concentration was one or two *L. donovani* promastigotes per sand fly blood-meal. The development of *L. donovani* was similar in both *P. orientalis* colonies; hence, the absence of visceral leishmaniasis in non-endemic area Melka Werer cannot be attributed to different susceptibility of local *P. orientalis* populations to *L. donovani*.

## Author Summary

*Phlebotomus orientalis* is the main vector of *Leishmania donovani* in East Africa and is, therefore, a sand fly species of high importance. We studied various properties of *P. orientalis* populations from both endemic (Addis Zemen) and non-endemic (Melka Werer) areas in Ethiopia. We successfully demonstrated the ability of laboratory colonies arising from these populations to crossbreed by obtaining 1^st^ and 2^nd^ generation hybrid progeny. Hybrids had similar or even higher fecundity than parental colonies. Comparison of the populations by sequencing of two genes (cytB and COI) and by RAPD (a multilocus method) revealed no genetic differences. We demonstrated that both populations are highly susceptible to experimental infection with *L. donovani* and even small numbers of parasites are able to initiate heavy infections in *P. orientalis* females. As the development pattern of *L. donovani* was similar for females from both colonies, we deduce that the absence of visceral leishmaniasis in the non-endemic area of Melka Werer cannot be attributed to different susceptibility of local *P. orientalis* populations to *L. donovani*.

## Introduction

Visceral leishmaniasis (VL, kala-azar) caused by the protozoan parasite *Leishmania donovani* is a deadly disease occurring mainly in the Indian subcontinent and Africa. In Africa, VL is endemic in the eastern part of the continent; the Horn of Africa and adjacent countries, namely Sudan, South Sudan, Kenya, Somalia, Uganda, Erithrea and Ethiopia. In Ethiopia, the main endemic areas are located in the lowlands of the southwestern Ethiopia (e.g. Omo river plains and Segen/Woito valleys) and Metema-Humera plains in the northwest [1]. Three sand fly species, *Phlebotomus orientalis, P. celiae* and *P. martini* have been implicated as vectors [2], [3]. *Phlebotomus celiae* Minter and *Phlebotomus martini* Parrot (both belonging to the subgenus *Synphlebotomus*) are limited to the south of the country, often being associated with termite hills, which provide suitable breeding sites. In the rest of Ethiopia, however, *P. (Larroussius) orientalis* seems to be the only vector.

Most biological information regarding habitat, seasonality and feeding preferences of *P. orientalis* was acquired thanks to demanding field studies in Sudan [4], [5], [6].The distribution of this species seems to be affected by the vegetation type, with preference for Acacia – Balanites forests and cracks of black cotton clay soil [7], [8], [9]. Additional important information, like actual breeding sites of this species, remains unknown. Despite several attempts of colonization of this species [10], [11] the life cycle and behaviour of *P. orientalis* in laboratory colonies has not been reported in detail and *P. orientalis* has a reputation of being difficult to colonize and maintain.

In this study, we focused on *P. orientalis* from two geographically distant Ethiopian localities, Addis Zemen (AZ) and Melka Werer (MW). Addis Zemen is located in the highlands of the Amhara Region in northwestern Ethiopia at altitude of 1800–2000 m where in 2005 and 2008, outbreaks of VL resulted in 2,500 cases and initially a very high mortality [12]. On the other hand, Melka Werer is a non-endemic area situated in Awash National game reserve in Rift Valley at an altitude of approximately 800 m, 200 km East of Addis Ababa.

Here, we compare individuals of both colonies by Random Amplification of Polymorphic DNA (RAPD) and sequencing analysis. The two populations were also tested for ability to produce viable hybrids in cross-mating studies. Different biological aspects of the two colonies found during the study allowed us to optimize the conditions for laboratory maintenance of both *P. orientalis* colonies, which appeared to be a fundamental prerequisite for the major goal of this work: experimental infections and comparison of susceptibility of both colonies to infections with *L. donovani*.

## Materials and Methods

### Ethical statement

Animals were maintained and handled in the animal facility of Charles University in Prague in accordance with institutional guidelines and Czech legislation (Act No. 246/1992 coll. on Protection of Animals against Cruelty in present statutes at large), which complies with all relevant European Union and international guidelines for experimental animals. All the experiments (including sand fly feeding) were approved by the Committee on the Ethics of Laboratory Experiments of the Charles University in Prague and were performed under the Certificate of Competency (Registration Number: CZU 327/99, CZ 00179). All samples were anonymized.

### Rearing sand fly colonies and life-cycle analysis

Both of *P. orientalis* colonies Addis Zemen (AZ) and Melka Werer (MW) were established in 2008 and reared for about ten generations at the Aklilu Lemma Institute of Pathobiology, Addis Ababa University, Ethiopia. For larval food, dried and ground hyrax faeces were used, females were fed on rabbits. Both the larvae and the adults were kept at 26°C. After transfer to Prague the sand flies were adapted to the conditions and the larval food routinely used in our laboratory [13]. Briefly, the larvae of both colonies were fed on a composted mixture of rabbit faeces and rabbit pellets. The suitability of autoclaved and non-autoclaved larval food was tested and compared. Adult sand flies were maintained on 50% sugar solution at 26–27°C. In the first generation after arrival to Prague, females were offered a blood-meal on rabbit or human arm (co-author PV served as volunteer), and within several generations they were adapted to feeding on anesthetized mice. The life-cycle details (length of egg development, each larval instar etc.) were collected from 12168 (AZ) and 8751 (MW) ovipositing females and recorded for over 20 months. Data monitoring the effect of nutrition on the life cycle of two *P. orientalis* colonies originate from the offspring of about 4,600 ovipositing females (2,200 MW and 2,400 AZ) during a three month period.

### Hemoglobin assay for measuring the blood-meal size

Due to massive prediuresis during bloodfeeding the classical weighing of bloodfed sand fly females leads to underestimation of the volumes of bloodmeals [14]. Therefore, the colorimetric method developed by Briegel *et al.* [15] for measuring the hemoglobin concentration in blood-fed mosquitoes was adopted. Females of *P. orientalis*, 3–6 days old, were fed through a chick-skin membrane on rabbit blood. Individual midguts of blood-fed females were dissected 1 h after blood-feeding, transferred to tubes containing 200 µl 0.15 mM NaCl and homogenized. Gut homogenates (50 µl) or diluted rabbit blood (5 µl rabbit blood/1000 µl 0.15 mM NaCl) were mixed with 200 µl of Drabkin's reagent (Sigma) in the dark for 30 min. Absorbance was measured in 96-well plates in doublets at 540 nm. Human hemoglobin (Sigma) in concentrations from 3.1 to 100 µg/well was used as standard. The bloodmeal volume was calculated from 40 midguts of fully bloodfed *P. orientalis* (MW) females.

### Cross-mating study

For the cross-mating study we slightly modified the method described by Dvorak *et al.* [16]. Briefly, individual pupae from each parental colony were separated into glass vials to obtain virgin adult flies. Virgin females from one colony were grouped with virgin males from the other colony (MW male/AZ female = Hybrids 1, AZ male/MW female = Hybrids 2) in an approximate 1∶1 ratio of sexes and allowed to feed on a human arm (PV served as a volunteer). Blood-fed females were separated and five days post blood-meal (PBM) transferred to moist oviposition pots to lay eggs. The egg production of hybrids was compared with both parental colonies (20 ovipositing females in each group). The parental and hybrid colonies were reared under identical conditions and their developmental life cycles were recorded (see Table 1). Adult F1 hybrids were used for F2 brother-sister mating to verify that F2 progeny were viable and develop similarly to parental lines.

**Table 1:** Life-cycle of two Ethiopian *P. orientalis* colonies and their hybrid F1 and F2 progeny.

			Life cycle in days PBM*							Egg production**		
			Eggs	Larvae			Pupae	Adults		Host	Eggs	
				L1	L2	L4		From	To		Total	Mean per female
Parental colonies***	AZ	mean	6.5	13.5	19.1	28.4	36.9	46.6	105.3	mouse	544	27.2
		range	5–9	11–19	16–29	23–34	31–47	39–69	61–147	human	975	48.75
	MW	mean	7.9	14.9	20.6	28.3	35.3	45.5	83.9	mouse	641	32.05
		range	4–12	12–20	18–24	24–32	29–41	40–52	54–110	human	693	34.65
Hybrids 1 ♂MW/♀AZ	F1		7	14	18	25	30	39	91	human	852	42.6
	F2		7	14	18	25	31	42	nd	human	846	42.3
Hybrids 2 ♂AZ/♀MW	F1		7	14	18	25	30	39	91	human	806	40.3
	F2		7	14	18	25	31	42	nd	human	812	40.6

* Days represent an interval between the female took a bloodmeal and the first offspring reached the respective instar.

** In the egg production study 20 ovipositing females were used in each group.

*** In the parental colonies the life cycle data were collected from 12,168 (Addis Zemen, AZ) and 8,751 (Melka Werer, MW) ovipositing females within the period from VIII/2010 to IV/2012. Each cell contains the mean and the range of values.

### Genetic analyses

The two *P. orientalis* colonies were compared by RAPD and by DNA sequencing of two mitochondrial genes, cytochrome B (cytB) and cytochrome oxidase I (COI). For RAPD analysis, eight specimens from each colony (four males and four unfed females) were selected randomly. Two other sand fly species were added into the analysis as outgroups: *Phlebotomus* (*Larroussius*) *tobbi* Adler and P*hlebotomus* (*Phlebotomus*) *bergeroti* Parrot. DNA was extracted using High Pure PCR Template Preparation Kit (Roche, France). Of 60 decamer random primers previously tested (OPA 1–20, OPD 1–20, OPF 1–20, by Operon Technologies Inc, USA), five were used: OPE16, OPI 12, 13, OPL5, OPO20. The PCR reaction was subjected to 45 amplification cycles in 25 µl volumes, with a temperature profile: 94°C for 1 min, 35°C for 2 min and 72°C for 3 min. An initial denaturation step of 94°C for 4 min and a final extension step of 72°C for 10 min were added. After PCR amplification, electophoretic bands were transformed into a binary matrix and genetic distances were computed from Nei-Li's coefficient of similarity [17]. Phylogenetic trees were constructed by the unweighted pair-grouping analysis (UPGMA) [18]. PC program FreeTree [19] was used for computations of genetic distances and construction of trees.

For sequencing analysis COI and a part of cytB genes were chosen. Templates for direct sequencing were amplified by PCR in a 50-µl volume using primers and conditions previously published [20], [21]. PCR products were sequenced in both directions using the same primers as for the DNA amplification on 3100 Avant Genetic Analyser (Applied Biosystems, USA). All PCR products were cleaned by QIAquick PCR Purrification Kit (Qiangen, Germany) prior to the sequencing. Obtained DNA sequence data were compared with those in the GenBank database. The sequences were aligned using ClustalX 1.81 and the resulting alignment was manually edited by BioEdit.

### Experimental infection of *P. orientalis*

Two *L. donovani* strains, GEBRE-1 (MHOM/ET/72/GEBRE1) and GR374 (MHOM/ET/2010/DM-1033) originating from VL patients in northern Ethiopia and kept in cryobank of the Department of Parasitology, Charles University were used for experimental infection of *P. orientalis*. Parasite strains were maintained at 23°C on medium 199 (Sigma) supplemented with 10% fetal calf serum (Gibco), 1% BME vitamins (Sigma), 2% human urine and amikin (250 µg/ml). Females of both colonies (∼–7 day old) were fed through a chick-skin membrane on a suspension of promastigotes (from 4-days-old *Leishmania* culture) mixed 1∶10 with heat-inactivated rabbit blood (Bioveta, Ivanovice na Hane, Czech Rep.). If not stated otherwise, an infective dose of 10^5^ promastigotes per ml of blood was used. To test dose-dependent differences in *Leishmania* development, GR374 cultures were used at the following concentrations: 2×10^3^, 2×10^4^, 10^5^ and 5×10^5^ promastigotes/ml of blood. Furthermore, the accurate number of parasites ingested by individual females (N = 8) was determined using Q-PCR immediately after the experimental feeding (details below).

Blood-fed females were separated immediately after feeding and kept at 26°C with free access to 50% sugar solution. One group of females was dissected for microscopical observations at different intervals PBM, the second group was placed into the plastic tubes filled with 100 µl of elution tissue buffer (from DNA isolation kit) on day 0 and 10 PBM and stored at −20°C for the following *Leishmania* DNA extraction, see below.

On days 2, 5–6, 8–11 PBM females were dissected in drops of saline solution. The individual guts were checked for presence and localization of *Leishmania* promastigotes under the light microscope, special emphasis was given to colonization of the stomodeal valve as the prerequisite for successful transmission [for review see 22]. Levels of *Leishmania* infections were graded into four categories according to Myskova *et al.* [23]: negative, light (<100 parasites/gut), moderate (100–1000 parasites/gut) and heavy (>1000 parasites/gut). Data were evaluated statistically by means of χ2 test using the S-PLUS 2000 program.

The number of *Leishmania* promastigotes in individual females was estimated by Q-PCR the SYBR Green detection method (iQ SYBR Green Supermix, Biorad, CA). The total DNA was isolated using a High Pure PCR Template Preparation Kit (Roche, Mannheim, Germany) according manufacturer's instruction. Kinetoplast DNA was chosen as the molecular target with primers described by Mary *et al.* [24]. Q-PCR was calibrated using serial dilutions of *L. donovani* DNA extracted from known number of promastigotes. Two microliters of eluted DNA was used per individual PCR reaction - 3 min at 95°C followed by 45 cycles of: 10 s at 95°C, 10 s at 56°C, and 10 s at 72°C. Results from Q-PCR were statistically evaluated using Kruskal-Wallis H-test.

## Results

### Life cycle of *P. orientalis* and differences between colonies

**Figure 1:**
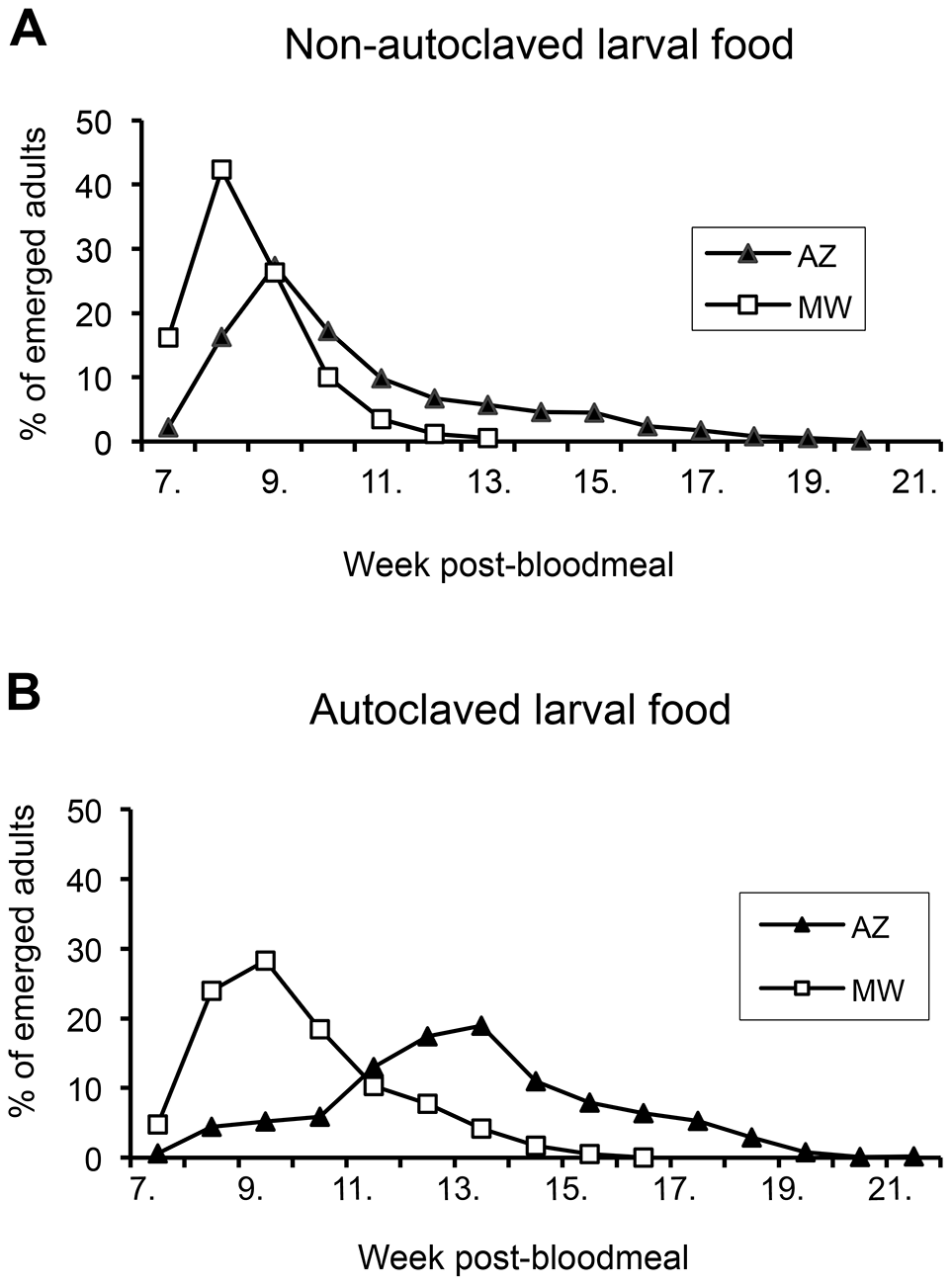
Effect of nutrition on the life cycle of two *P. orientalis* colonies. Data originate from the offspring of about 4,600 ovipositing females (2,200 MW and 2,400 AZ) during a 3 month period. **1A**: On the non-autoclaved food the number of adults emerging from pupae peaked on week 8 PBM in MW, and week 9 PBM in AZ. All individuals completed the life cycle within 13 and 20 weeks for MW and AZ, respectively. **1B**: On the autoclaved food the life cycle was prolonged and the larval growth appeared less synchronized in both colonies. The impact was more significant in the AZ colony: emergence of AZ adults peaked on week 13 (four weeks later than on non-autoclaved food).

The developmental data of both *P. orientalis* colonies are summarized in Tables 1 and Figure 1. The life cycle beginning with egg development in blood-fed females to eclosion of the adult sand fly (including egg, larval and pupal stages) ranged from seven to sixteen weeks in MW and from seven to twenty-one weeks in AZ (Figures 1A, B). In contrast to most other sand flies maintained in our laboratory *P. orientalis* larvae and adults (including blood-fed females) prefer relatively high humidity. However, AZ and MW colonies differ in humidity demands during pupation: while MW pupae concentrated close to the upper edge of the rearing pot, the AZ larvae pupated mainly in the substrate on the bottom of the pot. Different pupation strategies might reflect dissimilar humidity demands of the two *P. orientalis* populations adapted to different microclimatic conditions.

Development of both colonies was affected considerably by the quality of larval food. On non-autoclaved food the emerging adults peaked at eight and nine weeks PBM for MW and AZ, respectively, and most of the adults (>90% in MW and >60% in AZ) emerged within ten weeks (Figure 1A). On autoclaved food the differences between colonies were more obvious as the development of AZ colony was significantly delayed. Peak of emerging offspring was nine and thirteen weeks PBM for MW and AZ colony, respectively. Only 16% of individuals of AZ colony achieved the adult stage within ten weeks PBM (Figure 1B). The quality of food affected mainly the fourth instar larvae where significant proportion of larvae stopped feeding and went into dormant phase, while the early larval stages were unaffected. In AZ colony, the non-synchronized larval development and tendency to diapause (predictive dormancy) occurred even on the non-autoclaved food. The growth of the L4 larvae was slightly improved by supplementation with TetraMin (aquarium fish food) (data not shown).

### Cross-mating study

Reciprocal hybridization crosses of both colonies resulted in successful mating and insemination, and produced viable F1 and F2 progeny. Hybrids had very high fecundity and developed successfully. In the F1 generation, the mean number of eggs per female was 42.6 and 40.3 for hybrids 1 (MW male/AZ female) and hybrids 2 (AZ male/MW female), respectively, and 42.3 and 40.6 in F2 generation. This egg production was even higher than in parental colonies (see Table 1). Immature larval stages of hybrids developed similarly or even faster than the parents. In both hybrid colonies egg development took 7 days and the whole life cycle from egg laying to eclosion from pupae lasted 32 days and 35 days in F1 and F2 generations, respectively (Table 1).

### Genetic analyses

**Figure 2:**
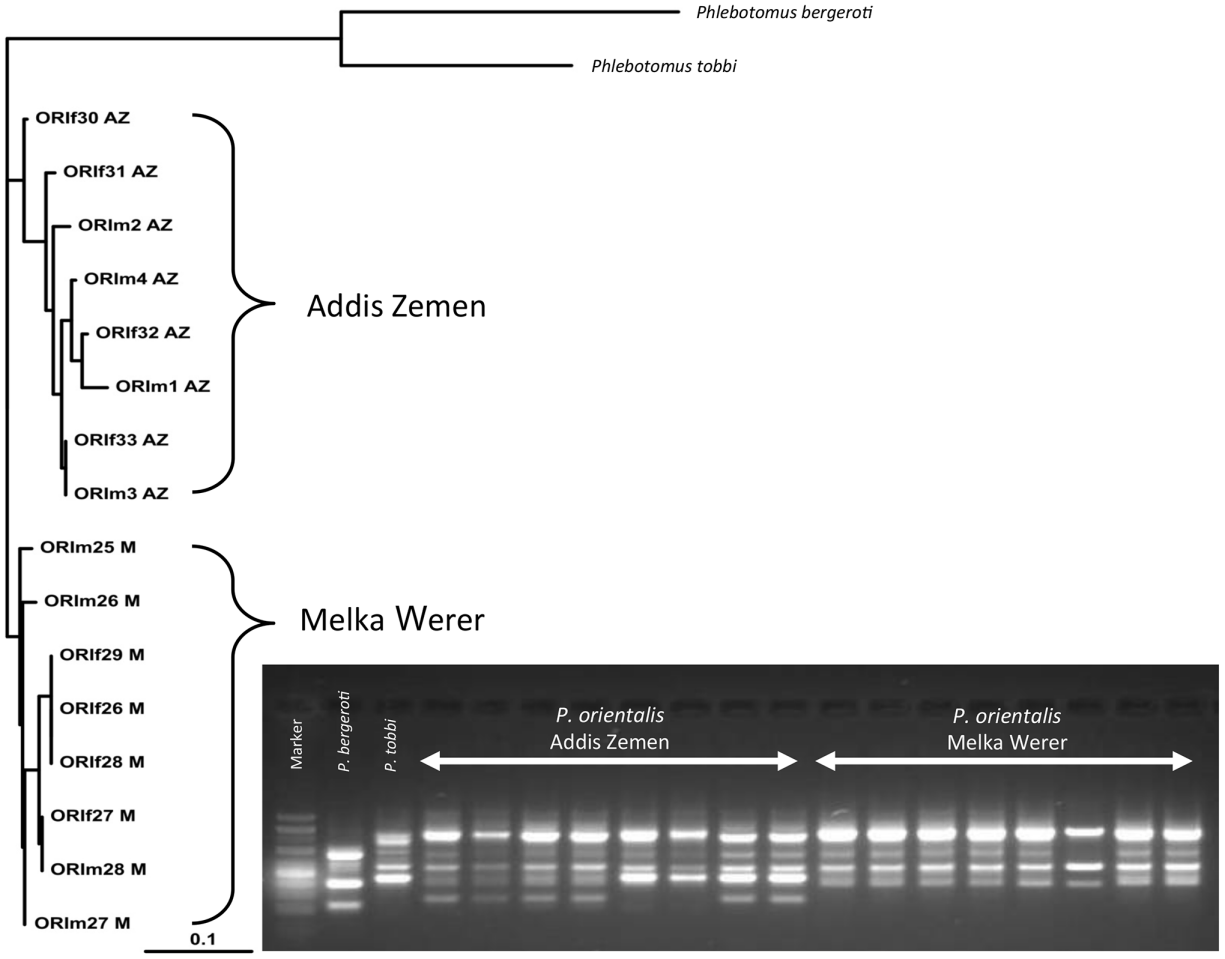
RAPD analysis of two *P. orientalis* colonies. RAPD analysis was based upon PCR results using five random primers (OPI12, 13, OPO20, OPE16, OPL5; in total 58 characters), electrophoretogram for OPL5 is shown as an example. Dendrogram was constructed by the Neighbor-joining method.

No morphological differences were found between *P. orientalis* colonies. Five decamer random primers were used for the RAPD analysis (Figure 2). A total number of 58 fragments, ranged from 100 to 1000 bp, were amplified. The band pattern given by amplification with each primer was reproducible and stable. The UPGMA analysis of these data revealed a position of two distinct clades, each containing specimens exclusively from one colony. None of the specimens fell into a clade of the other colony. A similar grouping pattern was also obtained by the neighbor-joining method (data not shown).

All analyzed CytB and CO-I sequences of several specimens belonging to both colonies were identical and no differences were observed. Sequences were submitted to GenBank (Accession numbers KC204965-KC204968).

### Development of *L. donovani* in *P. orientalis*

The susceptibility of both *P. orientalis* colonies to *L. donovani* was demonstrated first using GEBRE-1 strain. On day 2 PBM, parasites were located inside the intact peritrophic matrix as procyclic promastigotes and showing high intensity of infection in 75% of females. On day 6 PBM, all females had defecated and the infection rate was 78%. Elongate nectomonads were located mainly in the abdominal midgut while short promastigotes and metacyclic forms migrated forward to the thoracic midgut; in 62% of the infected females promastigotes colonized the stomodeal valve. Subsequently, on day 9 PBM, mature infection with high parasite burdens and colonization of the stomodeal valve were found in the majority (84%) of females (data not shown).

**Figure 3:**
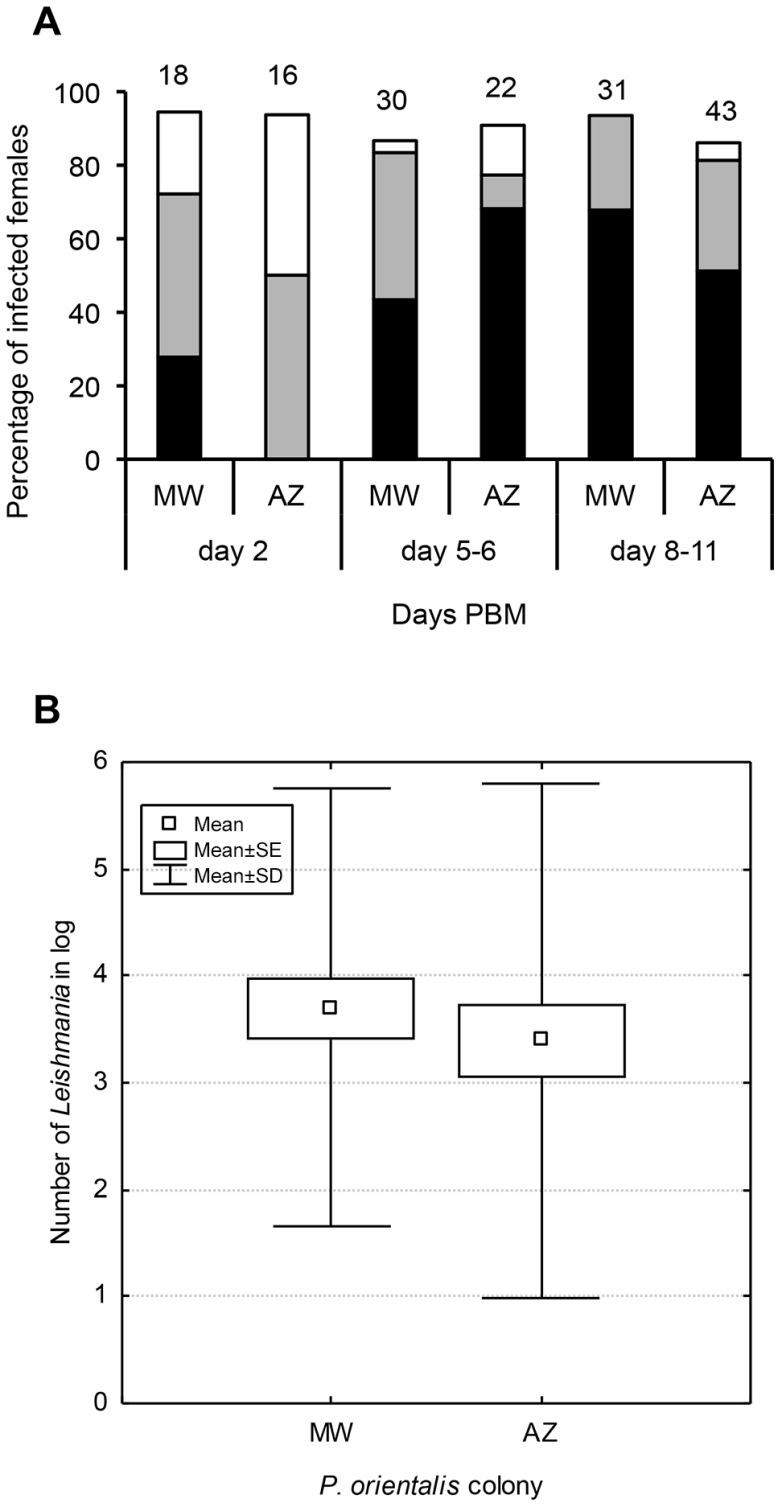
Development of *L. donovani* (GR 374) in females of two *P. orientalis* colonies. Sand flies were infected by feeding on a suspension of 10^5^ promastigotes/ml of blood and kept at 26°C. **3A**: Infected females of *P. orientalis* were examined microscopically 2, 5–6 and 8–11 days post-bloodmeal (PBM). The infection intensities were classified into three categories according to their intensity: heavy (more than 1,000 parasites per gut [black]), moderate (100–1,000 parasites [grey]) and light (1–100 parasites [white]). Numbers above the bars indicate the number of dissected females. **3B**: Parasite numbers from 40–50 individual females were quantified by Q-PCR targeted on amplification of *Leishmania* kDNA 10 days PBM.

Accurate determination of potential differences in vector competence of the two *P. orientalis* colonies was assessed by infections with *L. donovani* strain GR374. In the early stage of infection (on day 2 PBM) parasites developed similarly in both *P. orientalis* colonies (P>0.05). On day 5–6 PBM, the infection rates were high (around 90%) in both colonies and the intensity of infection was slightly higher in AZ colony (P = 0.048). Abundant metacyclic promastigotes (more than 50%) and colonized stomodeal valves were observed as early as 5 days PBM. On day 8–11 PBM, high infection rates (94% for MW and 86% for AZ) and similar intensities of infection were found in both colonies (P>0.05) (Figure 3A). Similarly, the Q-PCR revealed no significant differences (P>0.05) in total parasite numbers in sand fly midguts on day 10 PBM (MW vs. AZ; N = 50 engorged females) (Figure 3B).

**Figure 4:**
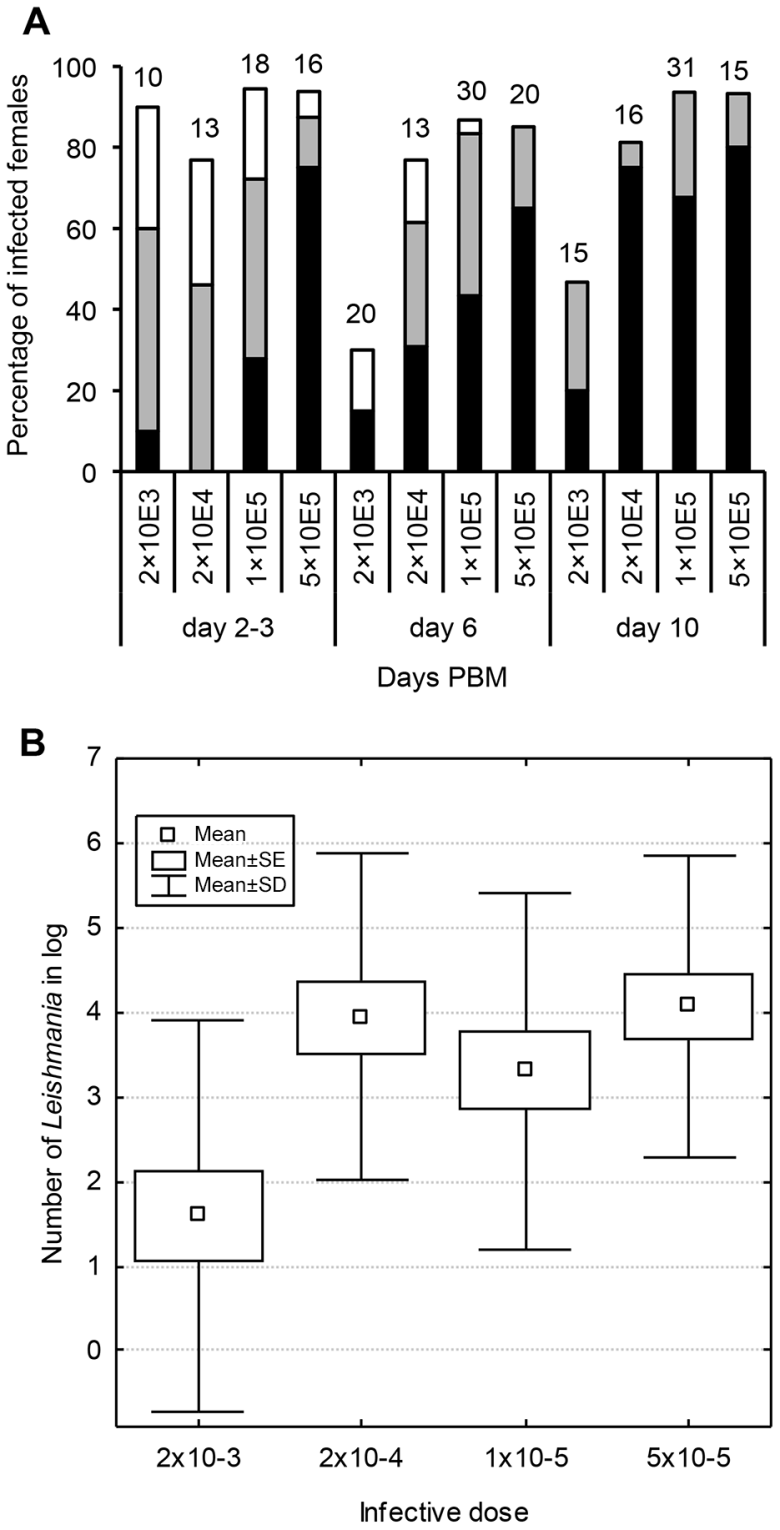
Effect of initial infective dose on development of *L. donovani* (GR 374) in *P. orientalis*. **4A**: Infected females of *P. orientalis* (MW colony) were examined microscopically 2–3, 6 and 10 days post-bloodmeal (PBM). The infection intensity was classified as described in Fig. 3. **4B**: Parasite numbers were determined using Q-PCR at 10 days PBM. Twenty females were used per group.

The effect of initial infective dose on total parasite numbers in sand fly gut during late stage infection was tested in *P. orientalis* (MW) infected by *L. donovani* (GR374) (Figure 4A,B). In fully bloodfed females of *P. orientalis* the average bloodmeal volume was 0.69 µl (SD = 0.1) ranging from 0.43 to 0.99 µl. It indicates that females infected of 5×10^5^, 10^5^, 2×10^4^ and 2×10^3^ promastigotes/ml of blood took on average 350, 70, 14 and 1–2 promastigotes, respectively. These results were confirmed by Q-PCR detecting accurate numbers of parasites from individual females immediately after blood feeding (data not shown). Despite the fact, that infection of sand flies was initiated with significantly different numbers of ingested promastigotes, the differences in infection rates were found only in group infected with 2×10^3^ promastigotes/ml. In this group the late stage infections (on days 6 and 10 PBM) were found only in 30–45% of females while in other three groups the positivity of females reached 75–95% (Figure 4A). However, the location of parasites during late stage infections was similar in all four groups tested and colonization of the thoracic midgut and the stomodeal valve was observed as early as on day 5 PBM. Even in the group infected with the lowest dose (2×10^3^ promastigotes/ml) numerous parasites colonizing the stomodeal valve were found in the majority (71%) of positive females on day 10 PBM.

The Q-PCR showed no significant differences in parasite loads at late stage infections (day 10 PBM) between groups of females infected with 5×10^5^, 10^5^ and 2×10^4^ promastigotes. In contrast, the significantly lower parasite loads (P<0.05) were found in group infected with 2×10^3^ promastigotes/ml of blood (Figure 4B); however, even this lowest dose was high enough to infect about 50% of females.

## Discussion

Sequencing analysis of cytB and COI genes as well as RAPD confirmed the high degree of similarity between the MW and AZ colonies originating in geographically distant areas and different altitudes. Despite this fact, obvious differences were found in certain life-cycle parameters of these populations.

The critical factor affecting larval development was the quality of larval food; autoclaved food resulted in a high proportion of dormant larvae and prolonged the generation time with the AZ colony being more sensitive to this change. Diapause of 4^th^ instar larvae has been described in some Palaearctic species, whereas species from warmer, wetter habitats are expected to diapause at the egg stage [25]. Our findings, as well study by Schmidt [26], proved the presence of diapause in the fourth larval stage in *P. orientalis* populations. The diapause and the non-synchronized larval development in the AZ population might be explained as an adaptation to more challenging natural conditions of the highland area, and probably assure that at least some of the population will survive through periods with challenging climatic conditions. A significant proportion of fourth instar AZ larvae diapaused despite of being maintained under a constant temperature of 27°C. This finding is in contrast with observations on other sand fly species where higher temperatures decreased the tendency of larvae to diapause [27].

The results of blood-meal analysis in females from endemic sites in Ethiopia showed bovines as preferred hosts of *P. orientalis* in natural conditions (about 92% of tested females) with a low proportion of females fed on humans [28]. In laboratory conditions an alternative bloodmeal source has to be adopted for the long term colonization. The AZ colony was less adaptable for substituting of blood-meal source than the MW colony. After arrival to the laboratory in Prague, females of both colonies were bloodfed on rabbits. MW females fed readily despite the initial small size of the colony and were adapted to anesthetized mice relatively easily within two or three generations (about six months). On the other hand, AZ females originally refused feeding even on rabbits and had to be offered a human arm. Adaptation for feeding on mice took more than ten generations (almost two years). To date, adaptation has not been 100% successful yet, and AZ females must be fed alternatively on rabbits and mice. Differences between the two colonies were also noted during experimental membrane feeding: AZ females were more reluctant to feed through a chick-skin membrane. Data on egg production seem to be in accord with requirements of AZ for blood source; AZ females fed on mouse produced less than 60% of eggs than those fed on human arm (see Table 1). For more robust conclusions a study on a larger sample would be needed.

The susceptibility of *P. orientalis* to *L. donovani* is the crucial factor for the epidemiology of visceral leishmaniases. Natural infections of *P. orientalis* with *L. donovani* were repeatedly reported from various foci in East Africa [1], [4], [11], [29], but only once in the south-west Ethiopia [30]. In Sudan, the susceptibility of *P. orientalis* to *L. donovani* has also been demonstrated by feeding on patients with kala-azar [10], [31] or by feeding infected blood through mouse-skin membranes [11]. These pioneering studies were, however, done using a limited number of *P. orientalis*.

In our study both tested strains of *L. donovani* developed very well in *P. orientalis* females and colonized anterior parts of the midgut and the stomodeal valve. Parasite development at 26°C was relatively fast as the presence of metacyclic promastigotes and colonization of stomodeal valve by haptomonads was observed already on day 5 PBM. On day 10 PBM, the infection rates in both colonies were very high (93% [MW] and 81% [AZ]) and the Q-PCR revealed that females from the two colonies did not differ in total numbers of parasites in their midguts.

The volume of *P. orientalis* blood-meals measured by hemoglobinometry was on average 0.7 µl of blood. This is about one half of the volume reported for *L. longipalpis* using the same technique [32]; the difference can be easily explained by body size as *P. orientalis* is a smaller sand fly.

Experimental infections revealed that even the lowest infective dose tested (2×10^3^ *L. donovani* promastigotes per ml of blood) was sufficient for high infection rates and successful establishment of late stage midgut development of this parasite in about 50% of females. Taking into account the average bloodmeal size of *P. orientalis* this concentrations is equivalent to infective dose between one and two *L. donovani* promastigotes per fly. This finding suggests extremely high susceptibility of *P. orientalis* for *L. donovani*; at present, the similar study using amastigotes is underway in our laboratory. Due to technical difficulties similar studies using amastigotes have not been performed yet in *P. orientalis*, however, in *L. longipalpis* Freitas *et al.* [33] demonstrated that promastigote-initiated *L. infantum* infections are fully comparable to amastigote-initiated ones.

In summary, this study describes in details behavioural and life-cycle parameters of two laboratory colonies of *P. orientalis* originating from Ethiopia and advances the knowledge of *P. orientalis* biology. We showed that demands for laboratory maintenance may significantly differ between two sand fly colonies of the same species. Therefore, the conditions of sand fly rearing should not be considered uniform and have to be optimized individually for each colony. Importantly, the study brings the first detailed description of *L donovani* development in *P. orientalis* under laboratory conditions. It proves that *P. orientalis* is a highly susceptible vector and only very low parasites are needed for establishment of experimental infections in this sand fly species. In view of our findings, we deduce that non-endemicity of visceral leishmaniases in Melka Werer cannot be explained by low susceptibility of local *P. orientalis* to *L. donovani*.
